# Impact of SGLT2 inhibitors on cerebrospinal fluid dynamics and implications for hydrocephalus management

**DOI:** 10.1172/JCI188584

**Published:** 2025-06-10

**Authors:** Nishanth S. Sadagopan, Rushmin Khazanchi, Rishi Jain, Amy B. Heimberger, Stephen T. Magill

**Affiliations:** Department of Neurological Surgery, Northwestern University Feinberg School of Medicine, Chicago, Illinois, USA.

**Keywords:** Aging, Neuroscience, Diabetes, Neuroimaging, Neurological disorders

**To the Editor:** Sodium/glucose cotransporter 2 (SGLT2) inhibitors (SGLT2-Is) are increasingly used for glycemic control, cardiovascular and renal protection, and weight loss in type 2 diabetes mellitus (DM) (1). DM incidence ranges from 15% to 18% in patients with idiopathic normal pressure hydrocephalus (INPH), increased from general populations (2). SGLT2 (SLC5A2) is expressed in the choroid plexus epithelial and ependymal cells in humans, potentially influencing cerebrospinal fluid (CSF) production (3). INPH is a syndrome affecting up to 3% of individuals 65 and older, characterized by dementia, gait impairment, and urinary incontinence (4). INPH is treated with ventriculoperitoneal shunts (VPSs), but no approved pharmacological treatments exist. We observed a reduction in ventricular size in a patient with INPH after initiating an SGLT2-I (case 2), prompting a review of our institutional cohort to assess the impact of SGLT2-Is on ventricular size (Supplemental Table 1; supplemental material available online with this article; https://doi.org/10.1172/JCI188584DS1). The Institutional Review Board approved this study.

Three patients with INPH with imaging before and after SGLT2-I treatment were identified. Radiographic changes in ventricle size were quantified using Evans Index (EI) and the callosal angle (CA) at the anterior commissure (AC), posterior commissure (PC), and splenium (5). The last brain imaging before SGLT2-I initiation was compared with the first scan after SGLT2 inhibition (Figure 1A). For patients with a programmable VPS, shunt settings remained unchanged between scans.

The mean age at INPH diagnosis was 74 years (SEM: 3.3). Two patients were male, and one was female. All patients underwent VPS placement and showed postoperative improvement in INPH symptoms. Each patient started SGLT2-I therapy after VPS placement (Figure 1B). The average time from VPS placement to pre–SGLT2-I scan was 11.4 months (SEM: 4.9), from pre–SGLT2-I scan to SGLT2-I initiation was 11.7 months (SEM: 2.2), and from SGLT2-I initiation to first post–SGLT2-I scan was 5.7 months (SEM: 1.8).

Only the CA at the splenium and EI were measurable in all patients before and after SGLT2-I therapy. One patient experienced dramatic ventricle size reduction, rendering the CA at the AC and PC unmeasurable because of ventricular collapse (Figure 1A, case 2). This patient required a VPS valve adjustment after imaging to reduce CSF drainage. The mean EI before SGLT2-I was 0.34 (SEM: 0.1), and after SGLT2-I therapy EI was 0.26 (SEM: 0.1; 2-tailed t test, P = 0.2597). The mean CA before SGLT2-I was 88.1° (SEM: 15.3), and after SGLT2-I therapy CA was 105.1° (SEM: 17.9; 2-tailed t test, P = 0.0225), with all patients showing increased CA (Figure 1C). Ventricular volume changes were analyzed via segmentation before and after SGLT2-I therapy (Supplemental Figure 1).

This study reports ventriculomegaly changes following SGLT2-I therapy in patients with INPH. The CA at the splenium increased by a mean of 17.0°, which is the only radiographic parameter that predicts improvement in INPH symptoms (5). Supplementary volumetric analysis verified reduced ventricular volume after initiation of SGLT2-I treatment (Supplemental Figure 1). Patients showing ventricular changes after VPS may have a greater propensity to respond further to SGLT2-I therapy, though a larger study is needed. Given potential CSF drainage interactions in patients with INPH after initiating SGLT2-I therapy, practitioners should monitor these patients closely.

SGLT2 expression in the choroid plexus epithelium (2), combined with these observations, suggests SGLT2 inhibition may modulate CSF production and ventricular size. Furthermore, SGLT2 is upregulated in brain tissue after traumatic brain injury (TBI), and up to 46% of intensive care unit–admitted patients with TBI develop hydrocephalus (6, 7). Thus, SGLT2 inhibition may be a rational strategy for the treatment of posttraumatic hydrocephalus.

All patients had a CT scan at least 3 months after VPS placement, without change in shunt drainage setting, so ventricular size is expected to be stable. However, this retrospective study cannot rule out a delayed decrease in ventricular size in response to VP shunting, though this is unlikely and a limitation of this study. While excess CSF production is a potential mechanism for INPH, its etiology is still unclear, with factors such as ependymal cilia dysfunction and impaired CSF absorption implicated. It is uncertain if SGLT2 inhibition functions in the brain similarly to its role in the kidneys. Alternatively, systemically administered SGLT2-Is could reduce ventricular size secondary to diuresis, as SGLT2 is highly expressed in the kidneys. However, no changes in serum electrolytes or kidney function were observed in these patients. Further research is required to determine the mechanism of SGLT2 action in the brain and ependyma and its impact on CSF dynamics. Recent reports have shown that glucagon-like peptide 1 receptor agonists can decrease intracranial pressure, but this mechanism also remains unknown, highlighting the importance of monitoring for off-target effects of novel medications that may affect and modulate CSF dynamics (8). These observations provide the foundation for prospective clinical trials to evaluate the efficacy, safety, and long-term benefits of SGLT2 inhibition in treating INPH or posttraumatic hydrocephalus alongside the rationale to investigate the mechanistic role of SGLT2 in modulating CSF dynamics.

## Supplementary Material

Supplemental data

Supporting data values

## Figures and Tables

**Figure 1:**
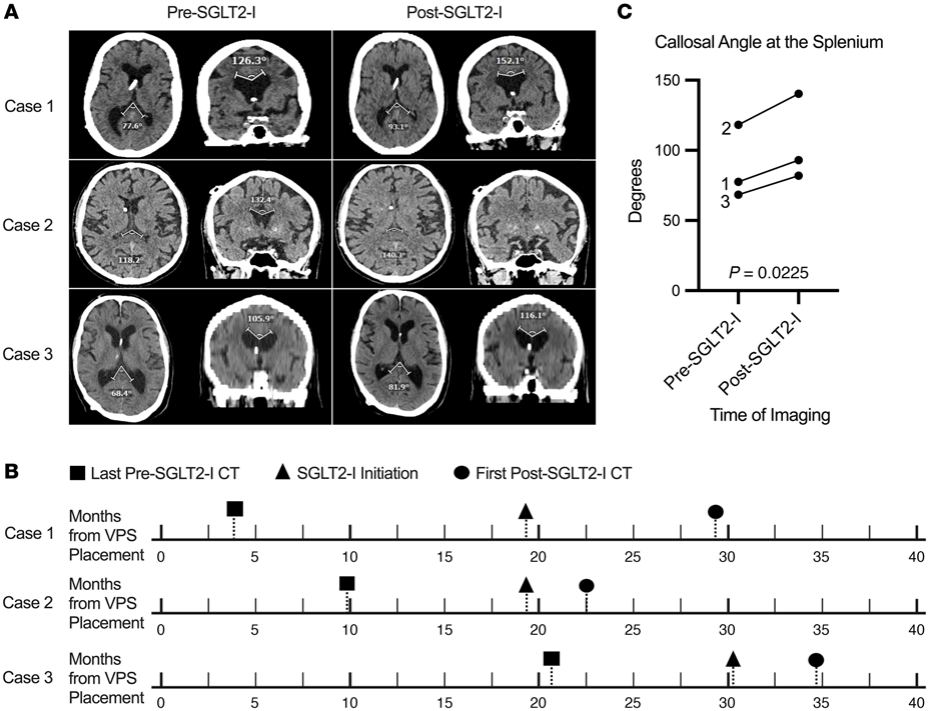
Effect of SGLT2 inhibition on ventricular size. (**A**) Axial and coronal CT showing callosal angle at the splenium and anterior commissure before (left) and after SGLT2 inhibition (right). (**B**) Imaging timeline after VPS placement and initiation of SGLT2 inhibition. (**C**) Quantification of change in callosal angle before and after SGLT2 inhibition (2-tailed *t* test, *P* = 0.0225).

## Version 1 06/10/2025

In-Press Preview

## Version 2 08/01/2025

Electronic publication

## References

[1] Tuttle KR (2023). Digging deep into cells to find mechanisms of kidney protection by SGLT2 inhibitors. J Clin Invest.

[2] Hudson M (2019). Comorbidity of diabetes mellitus in idiopathic normal pressure hydrocephalus: a systematic literature review. Fluids Barriers CNS.

[3] Chiba Y (2020). Sodium/glucose cotransporter 2 is expressed in choroid plexus epithelial cells and ependymal cells in human and mouse brains. Neuropathology.

[4] Williams MA, Relkin NR (2013). Diagnosis and management of idiopathic normal-pressure hydrocephalus. Neurol Clin Pract.

[5] Hattori T (2023). Correlation of callosal angle at the splenium with gait and cognition in normal pressure hydrocephalus. J Neurosurg.

[6] Oerter S (2019). Validation of sodium/glucose cotransporter proteins in human brain as a potential marker for temporal narrowing of the trauma formation. Int J Legal Med.

[7] Svedung Wettervik T (2022). Post-traumatic hydrocephalus - incidence, risk factors, treatment, and clinical outcome. Br J Neurosurg.

[8] Mitchell JL (2023). The effect of GLP-1RA exenatide on idiopathic intracranial hypertension: a randomized clinical trial. Brain.

