# Supplementary material for: Impact of SGLT2 inhibitors on cerebrospinal fluid dynamics and implications for hydrocephalus management
Source: J Clin Invest. 2025 Jun 10;135(15):e188584. doi: 10.1172/JCI188584 (PMC12321375; doi:10.1172/JCI188584)
Supplement: Supplemental data [file jci-135-188584-s259.pdf]

Supplementary Material

Methods

Sex as a biological variable

Our study examined male and female patients, and similar findings are reported for both sexes. However, the cohort size is limited and differences between sexes were not analyzed.

Statistics

We compared the changes in the EI and CA at the AC, PC, and splenium between pre- and post-SGLT2-I therapy with 2-tailed student's T-tests. For all analyses, statistical significance was defined as $p < 0.05$.

Study Approval

This study was granted approval by our Northwestern Institutional Review Board (#STU STU00218997) in Chicago, IL. All patients provided written informed consented to their surgical procedure and investigation for research purposes.

Data Availability

Data is available in the supplemental XLS files.

Author Contribution

Author order for NSS and RK was determined by coin-flip. STM conceived the project. NSS and STM collected and interpreted the radiographic and clinical data. RK conducted the statistical analysis. RJ, RK and STM conducted volumetric analysis. NSS and RK drafted the initial manuscript. NSS created the tables and figures. STM and ABH critically revised the manuscript and provided study oversight. All authors read and approved the final manuscript.

Acknowledgements

The authors would like to thank Dr. Asadollah Moshai, MD for his thoughtful clinical observations and discussions, which sparked this study.

Supplementary Table 1. Cohort Characteristics, n = 3

Variable	Value
Estimated Mean age at diagnosis [SEM]	76.0 [1.732]
Male sex, n [%]	2 [66.7%]
Estimated Mean BMI [SEM]	26.6 [1.423]
**Comorbidities**	
Diabetes, n [%]	3 [100%]
Hypertension, n [%]	3 [100%]
CHF, n [%]	0 [0%]
MI, n [%]	0 [0%]
COPD, n [%]	0 [0%]
**Presenting symptoms**	
Gait impairment, n [%]	3 [100%]
Cognitive impairment, n [%]	3 [100%]
Urinary incontinence, n [%]	3 [100%]
**iNPH Treatment**	
VPS Placed, n [%]	3 [100%]
**SGLT2 Inhibitor Therapy**	
Canagliflozin, n [%]	1 [33.3%]
Empagliflozin, n [%]	2 [66.7%]

Abbreviations: BMI: body mass index; CHF: congestive heart failure; MI: myocardial infarction; COPD: chronic obstructive pulmonary disorder; iNPH: idiopathic normal pressure hydrocephalus; VPS: ventriculoperitoneal shunt; SGLT2: sodium/glucose transporter 2.

Supplementary Figure & Legend.

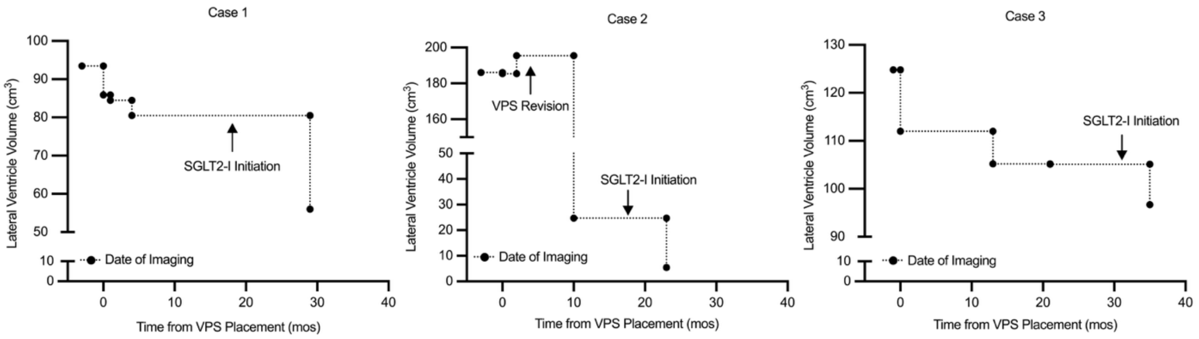

Supplementary Figure 1: Lateral ventricular volume (cm^3) graphed over time for each case, with timing of initiation of SGLT2-I treatment. The black dots indicate the timing of imaging in months since initial VP shunt placement at time zero. The pre-operative ventricular volume is shown to the left of time zero.
